# Supplementary material for: Neck circumference is a highly reliable anthropometric measure in older adults requiring long-term care
Source: PeerJ. 2024 Jan 31;12:e16816. doi: 10.7717/peerj.16816 (PMC10838066; doi:10.7717/peerj.16816)
Supplement: Supplemental Information 1 — ICC, intraclass correlation coefficient; CI, confidence interval; ECW/TBW, extracellular water/total body water. [file peerj-12-16816-s001.docx]

**Supplementary Table1. Intra-rater reliability for neck circumference measurement**

|  | n | ICC (1,1) | 95% CI |  |
| --- | --- | --- | --- | --- |
| Examiner A |  |  |  |  |
| All | 108 | 0.9995 | (0.9993–0.9997) | |
| Male | 66 | 0.9994 | (0.9990–0.9996) | |
| Female | 42 | 0.9991 | (0.9983–0.9995) | |
| Age (years) 65–74 | 19 | 0.9993 | (0.9981–0.9997) | |
| Age (years) 75≤ | 89 | 0.9996 | (0.9994–0.9997) | |
| ECW/TBW<0.40 | 68 | 0.9996 | (0.9993–0.9997) | |
| ECW/TBW≥0.40 | 40 | 0.9994 | (0.9989–0.9997) | |
|  |  |  |  | |
| Examiner B |  |  |  | |
| All | 108 | 0.9996 | (0.9995–0.9997) | |
| Male | 66 | 0.9994 | (0.9990–0.9996) | |
| Female | 42 | 0.9994 | (0.9989–0.9997) | |
| Age (years) 65–74 | 19 | 0.9996 | (0.9991–0.9999) | |
| Age (years) 75≤ | 89 | 0.9996 | (0.9994–0.9998) | |
| ECW/TBW<0.40 | 68 | 0.9997 | (0.9996–0.9998) | |
| ECW/TBW≥0.40 | 40 | 0.9993 | (0.9987–0.9996) | |

ICC: intraclass correlation coefficient; CI: confidence interval; ECW/TBW: extracellular water/total body water.
